# Effect of a high-fat diet on the rat bladder wall and bioactive action of Brazil nut oil

**DOI:** 10.1590/S1677-5538.IBJU.2018.0547

**Published:** 2019

**Authors:** Aline Costa de Souza, Carla Braga Mano Gallo, Magna Cottini da Fonseca Passos, Carolina Croccia, Glauciane Lacerda Miranda, Francisco José Barcellos Sampaio, Bianca Martins Gregório

**Affiliations:** 1 Unidade de Pesquisa Urogenital, Centro Biomédico, Universidade do Estado do Rio de Janeiro, Rio de Janeiro, RJ, Brasil;; 2 Departamento de Nutrição Aplicada, Instituto de Nutrição, Universidade do Estado do Rio de Janeiro, Rio de Janeiro, RJ, Brasil;; 3 Instituto de Nutrição Josue de Castro, Universidade Federal do Rio de Janeiro, Rio de Janeiro, RJ, Brasil

**Keywords:** Bladder, Nuts, Diet, High-Fat, Rats

## Abstract

High-fat diet-induced obesity is associated with metabolic disorders. The Brazil nut has bioactive substances and has been used to control the damage caused by obesity in several organs. The work intended to show the damage caused by high-fat diet in the bladder wall and if the Brazil nut oil added to the diet could ameliorate or reverse this effect. Sixty-day-old rats were divided into two groups: C (control, n = 30) and HF (high-fat, n = 30) diets. At 90 days, 10 animals of each group were sacrificed. The others were divided into 4 groups: C and HF (animals that maintained their previous diet, n = 10 for each group) and C / Bno and HF / Bno (animals whose control or high-fat diet was supplemented by Brazil nut oil, n = 10 for each group). Sacrifice occurred at 120 days, and the bladders were removed and analyzed. Epithelial height was increased in the HF compared to the C group. In contrast, the C / Bno had a lower epithelial height compared to the others. The percentage of collagen between the detrusor muscle fibers was significantly greater in C / Bno, HF and HF / Bno than in control group. The HF had a larger muscle fiber diameter than the C group, while the C / Bno presented lower values than the HF and HF / Bno groups. HF diets induced bladder wall damage. These changes in the rat's bladder wall were partially reversed by the Bno.

## INTRODUCTION

The number of overweighed and obese individuals is increasing in the World. Weight gain affects individuals of all ages, social strata and ethnic groups. Although the etiology of obesity is complex, several factors are involved, particularly changes in food patterns (1).

High-fat diet-induced obesity is associated with higher incidences of insulin resistance, hypertension, dyslipidemia, cancers and certain metabolic disorders (2). Concerning the urogenital system, our group, working with experimental animals, showed that a high-fat diet promoted obesity and affected different organs, including the testicle (3), prostate (4) and penis (5). Other studies in both experimental animals (6, 7) and humans (7) have shown that lower urinary tract dysfunctions are associated with obesity. Several debilitating urological symptoms associated with obesity could represent pathogenic as well as functional involvement of the bladder (8). Most of the studies focusing on obesity versus the urinary bladder have demonstrated physiological dysfunctions without pointing out the morphological changes that lead to these comorbidities. Although the morphological bladder wall components, mainly collagen and muscle, have not yet been investigated, they are the substrate from which problems affecting the bladder arise.

The Brazil nut (Bertholletia excelsa) comes from the Amazon region and has a complex matrix of bioactive substances such as selenium, alpha-tocopherol, phenolic compounds, folate, magnesium, potassium, and calcium. It is rich in monounsaturated fatty acids (MUFAs) and polyunsaturated fatty acids (PUFAs) (9, 10).

Whereas the properties and beneficial effects of the Brazil nut have already been reported in different studies on inflammation (9), endothelial dysfunction (11) and obesity (9) no data in the literature has been found concerning the Brazil nut oil (Bno) and its potential benefits on the urogenital system, particularly the bladder.

This study gives continuity to previous studies on high-fat diets and the urogenital system (3–5) by analyzing the bladder wall of rats fed with a high-fat diet to determine the effect on morphology. We also hypothesized that the extracted Bno, when added to the diet, could ameliorate high-fat diet-induced metabolic disorders related to the bladder components. So, the work intended to show the damage caused by high-fat diet in the bladder wall and if the Brazil nut oil added to the diet could ameliorate or reverse this effect.

## MATERIALS AND METHODS

### 

#### Study design and experimental diets

The experiments were approved by the Ethics Committee of the Institute of Biology of the State University of Rio de Janeiro, per the recommendations in Law no. 11.794, promulgated in November 2008 and in the standards set by the Journal of Physiology of the British Society of Physiology.

**Figure 1:**
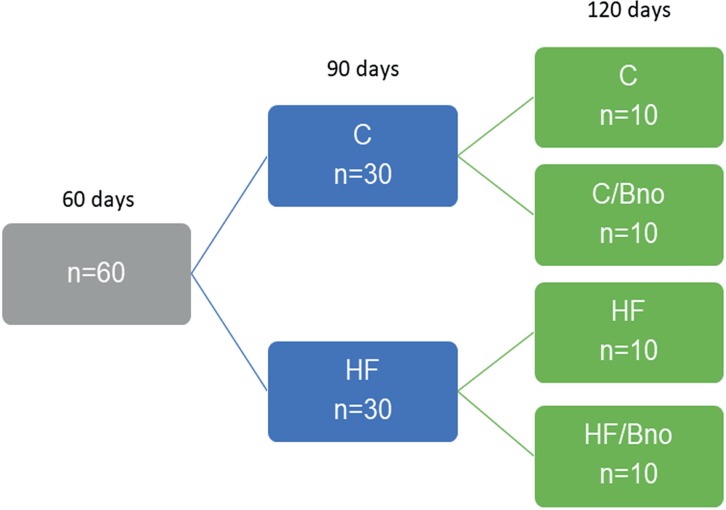
Study design. **C** (control diet at 90 and 120 days); **C / Bno** (control diet at 90 days and diet based on Brazil nut oil at 120 days); **HF** (high-fat diet at 90 and 120 days); **HF / Bno** (high-fat diet at 90 days and diet based on Brazil nut oil at 120 days). The animals were killed at 90 days and 120 days of age.

At 60 days of age, the animals were divided into two groups by experimental diet until 90 days of age: Control group (C, n = 30), fed with a casein-based diet and 4% soybean oil (normolipid) ad libitum (AIN 93-M - American Institute of Nutrition); high-fat group (HF, n = 30), fed with a casein-based diet and 4% soybean oil supplemented with 32% lard, ad libitum. At 90 days, 10 animals from each group were sacrificed. The 40 remaining animals were subsequently divided into 4 groups and were followed until 120 days of age (Figure-1).

1. Control group (C, n = 10), fed with a casein-based diet and soybean oil 4% (normolipid) (AIN 93-M), ad libitum.
2. Brazil nut oil control group (C / Bno, n = 10), fed with a casein-based diet and 4% soybean oil supplemented with 15% Bertholletia excelsa oil, ad libitum.
3. High-fat group (HF, n = 10), fed with a casein-based diet and 4% soybean oil supplemented with 32% lard, ad libitum.
4. Brazil nut oil high-fat group (HF / Bno, n = 10), fed with a casein-based diet and 4% soybean oil supplemented with 15% Bertholletia excelsa oil (AIN 93-M), ad libitum.

The food intake and body mass of the rats were monitored daily and weekly, respectively, until the end of the experiment. The experimental diets were formulated per the recommendations of the AIN-93 M for rodents (12). Each week, after extracting the lipids from the Bno, the diet ingredients were weighed and homogenized in a Hobart® (Professional Equipment for Industrial Kitchens. São Paulo, Brazil) industrial mixer with boiling water. The obtained mass was dehydrated in an oven ventilated at 50°C for 24 h and transformed into pellets and, after identification, was stored in a refrigerator until used. The diet composition is listed in Table-1. Bno was extracted by cold hydraulic pressing (Marconi®, model ME 098. Piracicaba, São Paulo, Brazil) at room temperature (31°C) at an initial pressure of 3 and a final pressure of 12 tons (Table-2).

**Table 1:** Composition of experimental diets.

Nutrients (g / 100g)	C diet	HF diet (lard)	HF diet (Brazil nut oil)
	%	%	%
Casein	14.00	14.00	14.00
Corn starch	62.07	29.95	46.95
Sugar	10.00	10.00	10.00
Minerais Mix	3.50	3.50	3.50
Vitaminas Mix	1.00	1.00	1.00
Fat (Soybean oil)	4.00	4.00	4.00
Fat (Lard)	-	32.00	-
Fat (Brazil oil nut)	-	-	15.00
Cellulose	5.00	5.00	5.00
Choline bitartrate	0.25	0.25	0.25
L-cystine	0.30	0.30	0.30
Energy (Kcal)	400.38	559.80	474.80

**C** = control diet; **HF** = High-fat diet

**Table 2:** Principal lipids from Brazil nut oil.

Fatty acid		Trivial name	Total amount (%)
**Saturated**			
	Tridecanoic Acid	-	4.103
	Hexadecanoic acid	Palmitic acid	13.763
	Octadecanoic acid	Stearic acid	10.109
**Monounsaturated**			
	Octadecanoic Acid	Oleic acid	32.838
	Nonadecanoic acid	-	35.728

At 90 and 120 days, the animals were anesthetized and euthanasied. The euthanasia was made with a mixture of ketamine (3.0 mg) and xylazine (0.6 mg) per 100 g of body weight intraperitoneally. After that, bladders were removed and fixed in 4% buffered formalin, following routine histological procedure.

#### Staining and morphometry

Five micrometer sections of bladder were stained with hematoxylin and eosin, Masson's trichrome, picrosirius and periodic acid-Schiff reagent (PAS) for morphometric quantification. Digital urinary bladder analyses were obtained using a microscope (BX51, Olympus, Tokyo, Japan) coupled to a digital camera (DP70, Olympus). The bladder epithelium height and muscle fiber diameter were evaluated using 60x and 100x objectives, respectively, and morphometrically analyzed by ImageJ Software (version 1.51k, Wayne Rasband, National Institutes of Health, USA). The amounts of detrusor muscle collagen and total collagen were determined using the point counting technique with an objective of 100x and 20x respectively and the same software. For all analyses, 5 fields per animal were considered (13, 14).

### Statistical analysis

Data were presented as the mean ± standard error mean (SEM). The differences between the groups at 90 days were calculated using an unpaired Student's t-test, and the group differences at 120 days were calculated using one-way and two-way ANOVA followed by Bonferroni's post-test. P < 0.05 was considered statistically significant (GraphPad Prism version 5.03 for Windows - GraphPad Software, San Diego, CA, USA).

## RESULTS

Daily food intake was lower in animals fed with the high-fat diet at 90 days (C: 18.45 ± 0.09 g, HF: 17.14 ± 0.38g, p < 0.0001) and 120 days (C: 33.40 ± 6. 56g, C / Bno: 31.46 ± 2.11g, HF: 17.00 ± 1.15g, and HF / Bno: 22.97 ± 1.72g, p < 0.0001) in relation to their respective controls. No differences in body mass were observed at 90 days. At 120 days, the group that received the HF diet, HF and HF / Bno was heavier (416.00 ± 49.51g and 486.60 ± 61.2g) than the other experimental groups (C: 381.30 ± 14.00g and C / Bno: 399.60 ± 60.96g).

**Figure 2:**
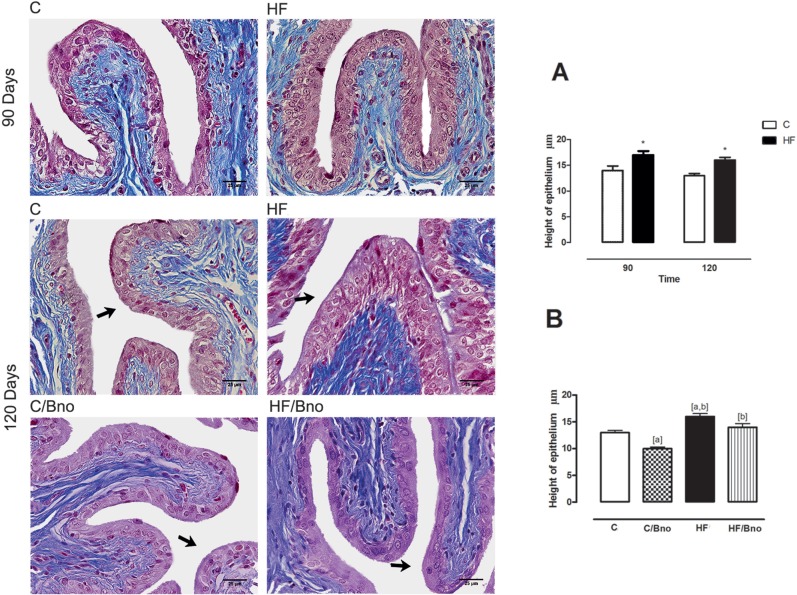
Image showing the height of the bladder wall epithelium (arrows). **A)** rats submitted to control and high-fat diets at 90 and 120 days, analyzed using a two-way ANOVA and t-test; [*], when ≠ C group. **B)** rats submitted to different diets at 120 days. Data were analyzed using an one-way ANOVA; [a], when ≠ **C**; [b], when ≠ C / Bno. Values represent the mean ± SEM of 10 animals per group, considering p < 0.05. Masson's trichrome, 600x.

Animals that received an HF diet showed a significant increase (+22%) in epithelial height at 90 days, and this difference was maintained at 120 days (Figure-2A). A two-way ANOVA showed that both diet type and the interaction between diet type and duration significantly affected the outcome, but duration alone did not. The HF group, which remained on the HF diet, presented a significant increase (+23%, p < 0.01) in the epithelial height compared to the C group. In contrast, the C / Bno group presented a lower epithelial height than C (-23%, p < 0.01) and HF groups (-38%, p < 0.0001 and -28%, p < 0.0001) (Figure-2B).

**Figure 3:**
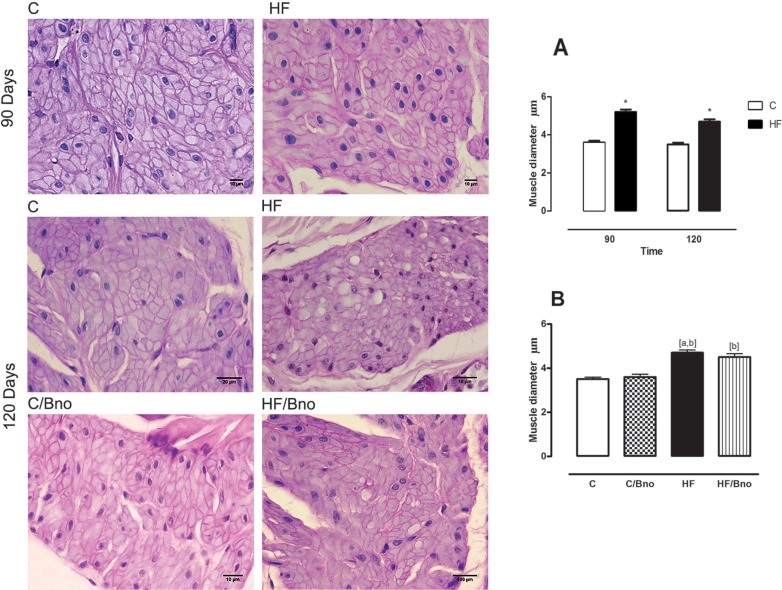
Image showing the diameter of the detrusor. **A)** rats submitted to control and high-fat diets at 90 and 120 days, analyzed using a two-way ANOVA and t-test; [*], when ≠ C group. **B)** rats submitted to different diets at 120 days. Data were analyzed using an one-way ANOVA; [a], when ≠ **C**; [b], when ≠ C / Bno. Values represent the mean ± SEM of 10 animals per group, considering p < 0.05. PAS, 1000x.

The animals that received an HF diet presented a significant increase in muscle fiber diameter at 90 days (Figure-3A) that was maintained at 120 days. A two-way ANOVA showed that there was a significant effect of the interaction between diet type and duration on the outcome, but there was not a significant effect from duration alone. Both groups receiving the HF diet had muscle fibers with a greater diameter (+34%, p < 0.001) in comparison to the control group (C) (Figure-3B). The C / Bno group presented lower values than HF and HF / Bno groups (-30%, p < 0.001).

**Figure 4:**
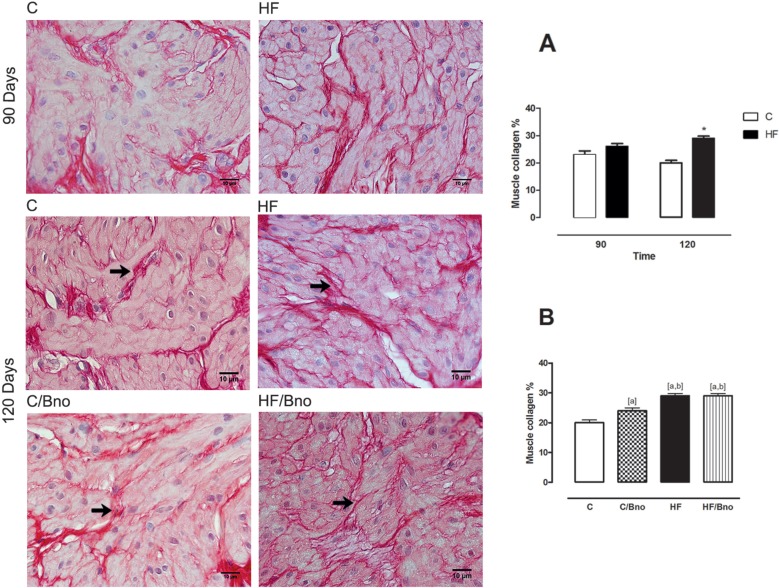
Image showing the collagen in the detrusor muscle (arrows). **A)** rats submitted to control and high-fat diets at 90 and 120 days, analyzed using a two-way ANOVA and t-test; [*], when ≠ C group. **B)** rats submitted to different diets at 120 days. Data were analyzed using an one-way ANOVA; [a], when ≠ **C**; [b], when ≠ C / Bno. Values represent the mean ± SEM of 10 animals per group, considering p < 0.05. Picrosirius red, 1000x.

At 90 days, the groups did not significantly differ in the percentage of collagen in the detrusor muscle, while at 120 days, the percentage of collagen in the HF group increased significantly (+30%) (Figure-4A). A two-way ANOVA showed that both diet type and the interaction between diet type and duration significantly affected the outcome, but duration alone did not. At 120 days (Figure-4B), both the high-fat diet (HF) and the nut oil diet (C / Bno and HF / Bno) groups had higher percentages of collagen in the muscle (+45%, p < 0.001; +17%, p < 0.001; and +45%, p < 0.001, respectively) than the C group. However, the C / Bno group showed a lower percentage of collagen than the HF and HF / Bno groups (-20%, p < 0.01). Concerning the percentage of total collagen present in the bladder, it was similar among the groups.

## DISCUSSION

The use of medicinal plants has shown interesting results in experimental research (15). Our results showed that the animals that received HF diets presented a higher body mass despite a lower food intake. This finding may be a consequence of the rat's dietary behavior, which, in the presence of a hyperenergetic diet, is adjusted for the amount of food ingested at the same energy value. Furthermore, despite eating less, the rats still gained more weight, which is due to the greater efficiency of the HF diet in accumulating adipose tissue (16).

Lower urinary tract dysfunctions are often associated with HF diets (6, 7). The intake of some fats, such as lard, negatively affects carbohydrate metabolism and maximizes the damage in different organs of the urogenital system (4, 5). The bladder, despite the important micturition problems that may be present, has not been sufficiently investigated when submitted to an HF diet.

Fatty acid metabolism is related to cellular functions and can be observed in cellular proliferation mechanisms. The HF diet likely caused circulating free fatty acids to mediate biological effects linked to cell proliferation, which triggered the increase in epithelial height observed in the present study (7).

The changes in the rat bladders caused by ingesting HF diets allowed us to verify whether Bno exerts its actions on the bladder wall components. The therapeutic effects of Brazil nuts have already been demonstrated in cardiovascular system (10, 11). However, the composition of Bno differs from chestnut composition since it changes while being pressed for oil manufacturing. Therefore, we cannot assume that the oil properties are similar to those of the chestnut.

In this work, the addition of Bno to control and HF diets significantly decreased epithelial height, showing a protective action that minimize the effects of lard. This effect is likely associated with the significant amounts of oleic acid (32.83%) and stearic acid (10.09%) in Bno, both important in the control of cell proliferation mechanisms (17). Supporting this rationale, studies have shown that urinary bladder cancer cells may have an increased lipid metabolism. Navarro-Tito et al. (2010) (17) found that administering oleic acid to non-tumor mammary epithelial cells does not induce metalloproteinase-9 secretion and controls cell proliferation better, whereas high levels of stearic and oleic acids are important indicators of bladder tumorigenesis (18).

The detrusor muscle in the bladder wall regulates bladder functions. Physiological studies have demonstrated the influence of HF diets on muscle fibers, showing impaired effects on mitochondrial functions including the uncoupling of oxidative phosphorylation and the decrease of endogenous antioxidant defenses (19). Concerning morphology, we found significant increases in muscle fiber diameter in the animals fed with the HF diet. However, unlike its effect on epithelial cells, Bno did not exert any effect on muscle fiber hypertrophy either when added to the control diet or when added to the HF diet. Most likely, palmitic and stearic acids counteracted the beneficial effects of oleic acid.

The HF diet also acts on the detrusor muscle to increase collagen production, which was significantly increased (30%) at 120 days. An anomalous increase in collagen can inhibit bladder contractility and electrical impulse conduction through the wall. This increase is observed in chronically obstructed bladders in adult men (20). These data support the morphological studies and explain the functional changes that are affected by increase in collagen production.

Collagen ensures tissue tensile strength (21). Diets based on animal fat appear to be linked to bladder stroma proliferation (22). HF diets are associated with increased FGF-ß (fibroblast growth factor beta) synthesis, which increases collagen production in the stromal compartment of the bladder (23). Surprisingly, collagen found in the mucosa and submucosa of the bladder wall did not increase, as was observed in the detrusor. Similarly, the addition of Bno did not interfere with this parameter. Possibly, the saturated fatty acids present in this oil could be induced to elevate cell death, reduce total antioxidant capacity, and augment lipid peroxidation. However, it remains unclear how this fatty acid influences collagen behavior (24).

The present work used the Brazil nut oil obtained after pressing which causes the loss of some properties of the seed. A group of animals fed with a diet in which the nut was crushed instead of pressed would be desirable and perhaps could produce more conspicuous results in relation to its protective effect. This experiment is being carried out in our laboratory to test this hypothesis.

In summary, high-fat diets are associated with bladder wall damage as increased epithelial height, muscle fiber diameter and detrusor collagen. These morphological changes in the bladder wall of the rats were partially reversed by the Brazil nut oil.
